# Stability of canine and feline cerebrospinal fluid samples regarding total cell count and cell populations stored in “TransFix®/EDTA CSF sample storage tubes”

**DOI:** 10.1186/s12917-020-02698-5

**Published:** 2020-12-17

**Authors:** Laura Meier, Regina Carlson, Jasmin Neßler, Andrea Tipold

**Affiliations:** Department of Small Animal Medicine and Surgery, University of Veterinary Medicine, Hannover, Germany

**Keywords:** Cerebrospinal fluid, CSF, Storage, TransFix®/EDTA CSF sample storage tubes, Flow cytometry, Leukocyte count, Cytospin assessment

## Abstract

**Background:**

Because of fast leucocyte degeneration in cerebrospinal fluid (CSF) laboratory examinations of CSF samples should be performed approximately within 30 min after withdrawal. This study examines the storage of canine and feline CSF samples in “TransFix®/EDTA CSF Sample Storage Tubes” (Cytomark, Buckingham, UK) for preventing leucocytes from degeneration, so that routine and flow cytometry examinations are feasible up to 3 days after sampling.

**Results:**

After storage in TransFix® tubes, leukocytes could not be adequately stained with Türk’s solution and differentiating between erythrocytes and leukocytes was cumbersome. In addition, the cell morphology could not be sufficiently assessed on cytospin preparations because of shrunken leukocytes and indistinct cell nuclei. In contrast, by flow cytometry, a significantly higher cell count was measured over the entire study period in the samples stored in TransFix® tubes compared to the untreated samples. The antibodies (AB) against CD3, CD4 and CD21, against CD11b and against CD45 showed a good binding strength and thus enabled a good differentiation of cell populations. However, after storage in the TransFix® tubes, monocytes were no longer detectable using an AB against CD14.

**Conclusion:**

Based on these results, “TransFix®/EDTA CSF Sample Storage Tubes” can be used for extended storage prior to flow cytometric analysis of lymphocytes and granulocytes in CSF samples but not for detecting monocytes. However, standard examinations, such as microscopic cell counting and morphological cell assessment should be performed on fresh CSF samples.

**Supplementary Information:**

The online version contains supplementary material available at 10.1186/s12917-020-02698-5.

## Background

For the diagnosis of diseases of the central nervous system (CNS), laboratory examination of cerebrospinal fluid (CSF) is a frequently used method [14, 16]. The analysis of CSF provides further information on changes in the CNS of a patient and has a good sensitivity for the detection of diseases [10].

The function of the CSF is to protect, support and nourish the CNS [9]. Physiologically, the CSF contains very few nucleated cells and no red blood cells, because the blood-CSF barrier inhibits unregulated entering of blood components in the CSF [10]. In several diseases, such as sterile and non-sterile inflammation, trauma, neoplasia or disc herniation an increased number of nucleated cells in the CSF is detected [23, 24]. The CSF also contains only a small amount of protein in healthy animals [10].

Routine CSF analysis includes microscopic white (WBC) and red blood cell (RBC) count and microscopic evaluation of cytospin preparations to differentiate the individual cell populations and thus to determine their percentage distribution [11, 14]. Furthermore, CSF samples can also be analyzed by flow cytometry for the distribution of individual cell populations, but this method of analysis is not used in routine examinations [11].

Because of the low protein content of the CSF, nucleated cells degenerate quite fast in cerebrospinal fluid, in particular granulocytes [24]. However, cell count and their percentage distribution is an essential part of the diagnosis of many diseases of the CNS [7]. Rapid cell degeneration after sampling in the case of delayed laboratory diagnostic examination can lead to a falsified test result. The majority of the cell degeneration occurs within 1 h after CSF sampling [7]. Furthermore about 31% of cells cannot be identified after a storage period of 24 h at a temperature of 4 °C and even more than 50% of cells are unrecognizable after a storage period of 48 h [2]. Based on these findings, it is currently recommended to examine the samples with a maximum delay of 30 min after CSF collection [24], in order to avoid false results. However, this might cause a problem in veterinary practices without an in-house laboratory. Due to the rapid lysis of the cells in the CSF, which has a low protein content, various options for extending the shelf life of the CSF samples after sampling have been tested. For example, the effects of adding protein sources like autologous serum or storage in the refrigerator were tested [2, 5, 7, 18]. Nevertheless, in none of the methods hundred percent of the cells were conserved, so by testing the use of TransFix®-solution another method for cell preservation in CSF samples should be found.

Currently, “TransFix®/EDTA CSF Sample Storage Tubes” (Cytomark, Buckingham, UK) are commercially available. According to the manufacturer’s introductions these tubes prevent cellular degradation in CSF over 72 h (manufacturer introduction, Cytomark, Buckingham, UK), thus enabling investigation at a later date. These tubes include the TransFix® stabilization solution, which contains a buffer, an aliphatic aldehyde and heavy metal salts in combination with ethylenediaminetetraacetic acid (EDTA) [8]. So far, these tubes are only available for research purposes, but good results have already been achieved in human medicine to stabilize cell populations in CSF samples for flow cytometric analysis. De Jongste et al. showed that 30 min after sampling of human CSF directly in TransFix® tubes the number of leukocytes in CSF diluted with TransFix®-solution was 1.4 times higher than in untreated CSF [8]. After a storage period of 18 h the number of leukocytes was even 2.3 times higher than in untreated CSF samples [8]. Especially the number of lymphocytes was higher at both time points, whereas there was no significant difference between the number of granulocytes in untreated CSF and in CSF with TransFix®-solution [8]. After storage in TransFix®-solution some cell characteristics may have changed [4, 8]. For example the light scatter properties of the leukocytes was different after storage in TransFix®-solution but this change had no disadvantages for the cell identification using flow cytometry [1]. The TransFix®-solution did not influence the key antigen expression patterns even if the signal in the flow cytometry was weaker from stabilized cells [1]. There are several further human studies using TransFix® tubes successfully for stabilization of cells in CSF specimens [15, 17, 21]. However up to now TransFix® tubes have not been tested in veterinary medicine for CSF specimens.

The aim of the current study was to test the use of “TransFix®/EDTA CSF Sample Storage Tubes” in veterinary medicine on canine and feline CSF samples for their use in standard examinations such as leukocyte count and cell morphology assessment, and for their use in flow cytometry. We hypothesize that using the “TransFix®/EDTA CSF Sample Storage Tube” provides an opportunity for longer shelf life and cell stability in CSF samples so that an examination within 72 h after CSF sampling is possible.

## Results

### Part 1: microscopic cell count and evaluation of cytospin preparations

**Fig. 1:**
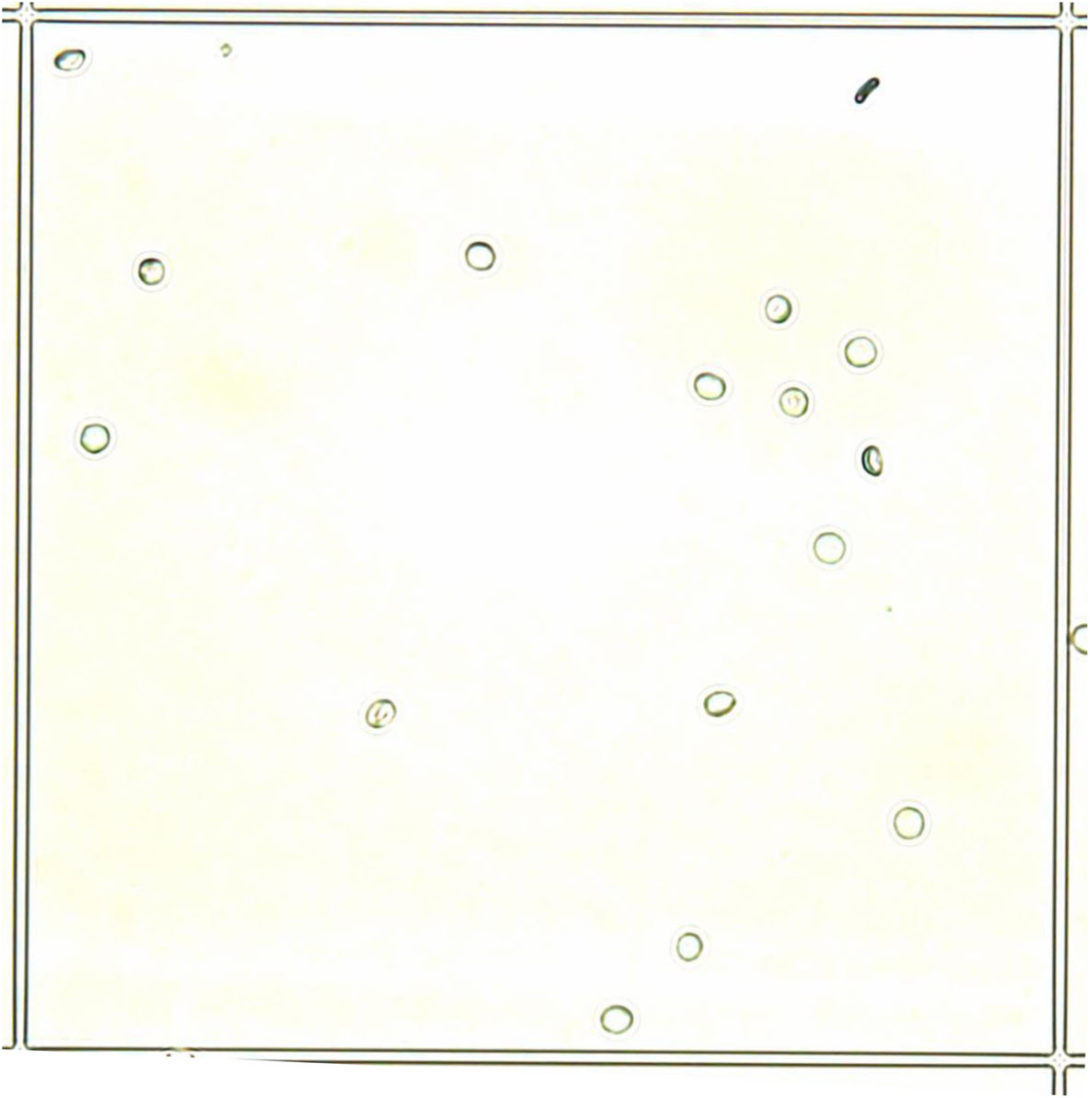
Leukocyte count on day 1 after storage in TransFix®-solution, 40x magnification

On day 0, the microscopic total nucleated cell count was simple and unequivocally determined, since the staining with Türk’s solution allowed a clear differentiation between WBC and RBC. On day 1 and 3, the difference between WBCs and RBCs in the untreated stored samples after staining with Türk’s solution was also evident in most of the samples (79/82, 96.3%), so that leukocyte and erythrocyte counts could be performed without any problems. In contrast, the distinction between RBC and WBC in the samples stored in TransFix®-solution on day 1 and day 3 was no longer possible. Using Türk’s solution, no cell structures could be stained and none of the visible cells showed a nuclear morphology. All visible cells seemed empty and only a rim was visible (Fig. 1). In addition, the cells varied in size from very small to large and differentiation of leukocytes and erythrocytes was not feasible. Many crescent-shaped cells were visible (Fig. 1). Due to the lack of dyeability and missing core structures, the cells could not be reliably differentiated from potential contamination or defect cell fragments. In three of the untreated samples, cell clumping was microscopically seen on day 1 and/or 3 and counting of WBCs and RBCs was not possible in these cases. No cell clumps were detected in any sample stored in TransFix®-solution.

Microscopic analysis of the individual cell populations on cytospin preparations also revealed a marked difference between the untreated CSF samples and the CSF samples stored in TransFix®-solution. The results can be found as supplementary information (Supplementary Material: Results Part 1). On day 0, nearly all cytospin preparations could be assessed well and the cells could be clearly differentiated microscopically. Only two cytospin preparations (2/43, 4.7%) revealed clumping of cells, so that the individual cell types in these aggregates could not be distinguished. On day 1 and 3, evaluation of the cytospin preparations of the untreated samples was still possible. In four of the untreated samples no cytospin preparations could be produced on day 3, because the amount of stored CSF was insufficient.

**Fig. 2:**
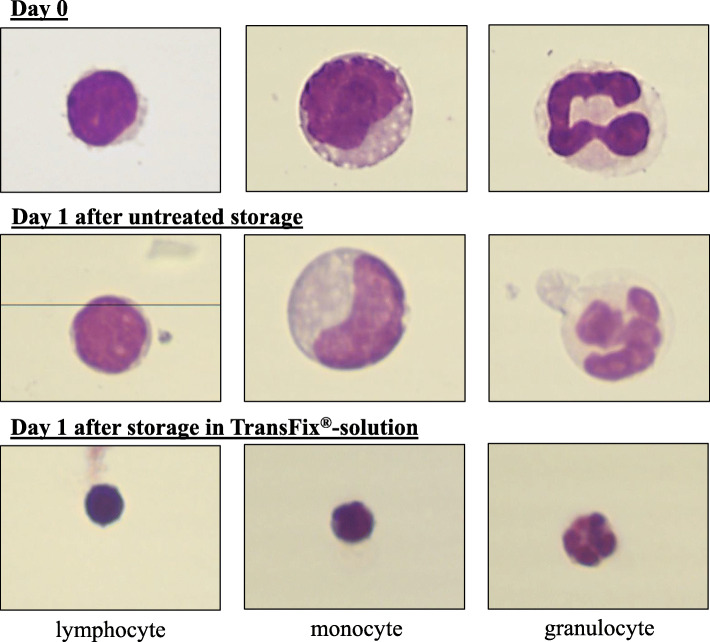
Comparison of the cell morphology on the cytospin preparations between day 0 and day 1 in untreated samples and storage in TransFix®-solution, 100x magnification

In contrast, the morphological differentiation of cell types in the cytospin preparations of the samples stored in TransFix®-solution was almost not feasible on day 1 and 3. The cells were significantly shrunken and did not differ in size (Fig. 2). The staining of the cells was very intense, in particular the lymphocytes were dark and uniformly stained without any recognizable nucleus or plasma space (Fig. 2). In some cells, a nucleus could be recognized by subtle changes at the macro and micro adjustments of the microscope. Neutrophilic granulocytes did not show their typical segmented cell nuclei any longer. The cytoplasm of the cells was barely recognizable, so that its color as well as possible vacuoles or granules were no longer clearly visible (Fig. 2).

### Part 2: flow cytometry

Table 1 shows the results of the descriptive statistics for the flow cytometry of leukocyte-spiked CSF pool samples divided by percentage and absolute cell counts for the total cell count, the dead cell proportion and all antibodies. The ANOVA revealed a significant main effect of the day, the type of storage as well as the interaction between the day and the type of storage on the total cell count (*p* < .0001). The total number of cells in the untreated samples decreased notably over the investigation period. In addition, the proportion of dead cells in the untreated samples increased significantly over the study period (*p* < .05). Despite the increase in percentage of dead cells, their absolute number, as well as the total number of cells, was continuously decreasing over the investigation period. In addition, macroscopically visible cell clumps were seen on days 1 to 3 in almost every of the untreated samples. In contrast to the untreated CSF samples, no significant decrease of the total number of cells was observed in the samples stored in TransFix®-solution (*p* > .05).

**Table 1:** Descriptive statistic results for the flow cytometry

	Mean (Standard Deviation) and Adj ***P***-Value						
		Absolute Count			Percentage		
	Day	Untreated	TransFix®	Adj P	Untreated	TransFix®	Adj P
Total Cell Count	0	71,176.85 (+/− 19,162.46)		–	–	–	–
	1	8944.25 (+/− 7364.30)	62,898.85 (+/− 15,425.89)	<.0001	–	–	–
	2	4711.95 (+/− 3629.14)	74,932.45 (+/− 24,044.26)	<.0001	–	–	–
	3	2886.10 (+/− 1619.68)	84,645.90 (+/− 29,320.20)	<.0001	–	–	–
Dead Cells	0	8448.05 (+/− 3559.04)		–	12.39% (+/− 5.25%)		–
	1	5939.05 (+/− 3132.97)	–	–	71.97% (+/− 11.43%)	–	–
	2	3975.25 (+/− 2163.75)	–	–	89.74% (+/− 9.88%)	–	–
	3	2845.83 (+/− 1504.27)	–	–	96.80% (+/− 3.29%)	–	–
CD3+ Cells	0	687.35 (+/− 577.97)		–	65.81% (+/− 25.01%)		–
	1	556.45 (+/ 520.38)	3058.35 (+/− 2051.57)	<.0001	70.63% (+/− 27.07%)	81.64% (+/− 13.02%)	.4275
	2	93.7 (+/− 64.21)	2695.35 (+/− 1808.93)	<.0001	75.88% (+/− 20.83%)	74.28% (+/− 13.65%)	1.000
	3	23.63 (+/− 14.94)	2829.1 (+/− 2267.99)	<.0001	92.27% (+/− 10.6%)	67.81% (+/− 17.56%)	.0456
CD4+ Cells	0	204.85 (+/− 158.67)		–	21.52% (+/− 9.41%)		–
	1	242.8 (+/− 249.16)	1531.0 (+/− 1172.43)	<.0001	32.18% (+/− 12.51%)	41.62% (+/− 12.86%)	<.0001
	2	41.05 (+/− 48.28)	1459.25 (+/− 1065.75)	<.0001	28.58% (+/− 13.0%)	40.77% (+/− 12.61%)	<.0001
	3	6.5 (+/− 3.3)	1609.15 (+/− 1301.27)	<.0001	29.5% (+/− 18.4%)	40.34% (+/− 11.9%)	.0015
CD11b + Cells (Histogram)	0	1674.25 (+/− 642.09)		–	93.09% (+/− 2.77%)		–
	1	443.88 (+/− 308.15)	25,116.65 (+/− 8471.01)	<.0001	86.57% (+/− 5.35%)	92.36% (+/− 5.0%)	.0417
	2	276.83 (+/− 257.68)	26,824.32 (+/− 11,113.98)	<.0001	85.3% (+/− 9.85%)	89.95% (+/− 6.68%)	.2348
	3	220.0 (+/− 197.99)	26,962.55 (+/− 8369.15)	<.0001	91.58% (+/− 2.38%)	87.93% (+/− 7.07%)	.9999
CD14+ Cells	0	1706.45 (+/− 3184.47)		–	46.43% (+/− 17.53%)		–
	1	93.17 (+/− 143.77)	–	–	27.69% (+/− 19.08%)	–	–
	2	–	–	–	–	–	–
	3	–	–	–	–	–	–
CD21+ Cells	0	605.45 (+/− 420.98)	–		22.4% (+/− 15.0%)		–
	1	156.9 (+/− 163.05)	742.5 (+/− 919.02)	.0001	15.33% (+/− 9.53%)	16.57% (+/− 13.67%)	1.000
	2	15.95 (+/− 15.15)	415.85 (+/− 304.61)	.0196	10.21% (+/− 6.16%)	10.85% (+/− 7.06%)	1.000
	3	2.67 (+/− 2.94)	308.95 (+/− 289.98)	.5413	5.79% (+/− 5.46%)	7.4% (+/− 7.087%)	.8135
CD45+ Lymphocytes	0	997.2 (+/− 695.28)		–	99.42% (+/− 0.85%)		–
	1	720.5 (+/− 543.46)	3685.85 (+/− 2312.73)	<.0001	99.78% (+/− 0.55%)	99.65% (+/− 0.38%)	.9999
	2	132.0 (+/− 99.21)	3503.9 (+/− 2003.84)	<.0001	98.81% (+/− 2.16%)	99.51% (+/− 0.46%)	.2478
	3	28.14 (+/− 19.57)	3898.60 (+/− 2636.39)	<.0001	99.76% (+/− 0.64%)	99.41% (+/− 0.61%)	.9962
CD45+ Granulocytes	0	1735.70 (+/− 675.16)		–	96.63% (+/− 2.16%)		–
	1	457.27 (+/− 403.02)	27,341.0 (+/− 9567.06)	<.0001	84.38% (+/− 10.96%)	99.64% (+/− 0.26%)	<.0001
	2	415.33 (+/− 330.73)	29,552.29 (+/− 12,476.98)	<.0001	94.04% (+/− 1.87%)	99.64% (+/− 0.23%)	.2415
	3	322.0 (1 sample)	30,340.79 (+/− 9239.22)	<.0001	83.42% (1 sample)	99.5% (+/− 0.37%)	.0135

The table shows descriptive statistics divided by percentage and absolute cell counts. In addition, the values for each antibody and for each day are indicated individually. The Adj *P*-values from the post-hoc test refer to the difference between the two types of storing on each day.

All samples stored in TransFix®-solution could be evaluated for the proportion of CD3- and CD4-positive lymphocytes. The ANOVA showed a significant main effect of the day and the type of storage as well as the interaction between the day and the type of storage on the absolute count of CD3- and CD4-positive lymphocytes (*p* < .0001). For the percentage of CD3- and CD4 positive lymphocytes the day and the interaction between the day and the type of storage showed a significant main effect in the ANOVA (*p* < .01) In contrast to the percentage of CD4-positive lymphocytes there was no main effect of the type of storage (*p* = .2596) in the ANOVA for percentage of CD3-positive lymphocytes.

For the antibody against CD21 all samples stored in TransFix®-solution could be evaluated without any problems. The ANOVA showed a significant main effect for the day (*p* < .0001), but there were no main effects for the type of storage and the interaction between the day and the type of storage (*p* > .05) on the percentage of CD21-positive lymphocytes. For the absolute count of CD21-positive lymphocytes the ANOVA showed a main effect of the day, the type of storage and the interaction between the day and the type of storage (*p* < .01).

The evaluation of the granulocyte population with the antibody against CD11b was first carried out by means of a histoplot. However, left-shift of the population of CD11b-positive granulocytes in the histoplot was observed in the untreated samples as well as in the TransFix®-stored samples during the study period. Therefore, samples were evaluated again using the histogram. The ANOVA showed a significant main effect of the day and the interaction of the day and the type of storage (*p* < .05) on the absolute count and the percentage of the CD11b-positive granulocytes. The effect of the type of storage was only significant for the absolute count of CD11b-positive granulocytes (*p* < .0001).

The antibody against CD45 was considered separately in the populations of lymphocytes and granulocytes. For the evaluation of the granulocytes in the samples stored in TransFix®-solution the histogram had to be used for four samples, but these were not included in the statistic evaluation. For the percentage of CD45-positive lymphocytes the ANOVA showed no main effect of the day, the type of storage and the interaction between the day and the type of storage (*p* > .05). On the contrary the ANOVA showed a main effect of all three variables for the absolute count of CD45-positive lymphocytes (*p* < .0001). For the absolute count and the percentage of CD45-positive granulocytes the day and the type of storage as well as the interaction between the day and the type of storage showed a significant main effect in the ANOVA (*p* < .0001).

In the untreated samples the antibody against CD14 showed a marked decrease in the proportion of evaluable samples in the following days due to a too low cell count. After storage in TransFix®-solution, this antibody could detect no monocytes in any sample and on any day neither with the histoplot nor with the histogram, so that the samples for this antibody were classified as not evaluable after storage in TransFix®-solution.

## Discussion

To our knowledge, this is the first study to use the TransFix® tubes for the stabilization of canine and feline CSF samples. In human medicine, there are positive effects for storage of CSF in the TransFix® tubes described amongst others by Almond et al. [1] and de Jongste et al. [8]. These results could only be partially confirmed in this study.

Because of the rapid degeneration of leukocytes in CSF samples [2, 20], several studies describe methods to extend the possible storage period of human and veterinary CSF samples between the sampling and the examination [2, 5, 7, 18]. However, the preservation method must not have an influence on further examination parameters of the CSF.

The results of this study have shown that the TransFix® tubes are not suitable for use in standard assays such as microscopic WBC and RBC count. After storage in TransFix®-solution leukocytes could not be adequately stained with Türk’s solution, so that no differentiation from the erythrocytes was possible. Due to the lack of dyeability and missing core structures, the cells could not be reliably differentiated from potential contamination or defect cell fragments, so the number of detected cells of the samples stored in TransFix®-solution on day 1 and 3 could be incorrectly higher than in the corresponding CSF sample on day 0. For the evaluation of the cytospin preparations the stabilization with TransFix®-solution provides only limited help because the microscopic evaluation of the cells was significantly impaired. The TransFix®-solution contains a buffer, an aliphatic aldehyde and heavy metal salts in combination with EDTA [8]. According to the safety data sheet of TransFix®-solution the aliphatic aldehyde is specified as paraformaldehyde which might lead to a deformation and shrinkage of the cells and thus prevents the dyeability of the leukocytes with Türk’s solution for the leukocyte count. Therefore we recommend that the leukocyte count and the morphological evaluation of the cells should be performed in a cytospin prepared within 30 min of sampling from fresh CSF samples before storage in TransFix®-solution.

For stabilization of cells in CSF prior to flow cytometric analysis, the TransFix®-solution can be used well. So far, flow cytometry has mainly been used for research purposes, but flow cytometry is already a widespread method in human medicine diagnostics [11]. In the future it could also have an increased usefulness in diagnostics in veterinary medicine. In particular, if the cells can be preserved in CSF samples and thus a time delay before the flow cytometry could not lead to falsified results, it would be easier to use it for diagnostics in the future. However, there are a few things to consider during evaluating the results of such conserved samples. The most important change to be mentioned is that no monocytes could be detected after storage in TransFix®-solution with the antibody against CD14. In contrast to the previous reported literature, which describes the successful use of the same antibody clone against CD14 in blood samples stored in TransFix®-solution [4], CD14 surface antigens no longer appear to be present on the canine monocytes in CSF after storage in TransFix®-solution. This does not necessarily mean that no monocytes can be preserved by the TransFix®-solution. The antibodies only bind to certain surface antigens and the surface antigens may lose their density or their typical structure and become unavailable for binding to the antibody after storage in the TransFix®-solution. This presumption is in contrast to previous reported results, which indeed describe a structural change of the cell membrane [4], but these changes are reported not to have any influence on the antigen density [4] and on the detectability of individual cell populations [1].

The measured total number of cells in the untreated samples and the samples stored in TransFix®-solution showed a marked difference between the two types of storage. The total number of cells in the samples stored in TransFix®-solution was on average 7 times higher than in the untreated samples on day 1. On day 2 and 3, the difference in the average number of cells between the samples of both types of storage continued to increase. Thus, the total number of cells in the samples stored in TransFix®-solution was almost 16 times higher on day 2 than in the untreated samples and on day 3 even about 29 times higher. These values exceed the values described in the literature [8]. De Jongste et al. described an average of 2.3 times higher leukocyte levels in the samples stored in TransFix®-solution compared to the untreated stored samples after an 18 h storage period [8]. After a storage period of 30 min after CSF sampling, a 1.4-fold higher leukocyte content was measured in the samples stored in TransFix®- solution than in the untreated samples [8]. For this value, no comparative values could be determined in this study, since the removal did not take place directly in the TransFix® tubes and the first comparison of both types of storage only took place on day 1. The factor many times higher compared to previous studies between untreated samples and samples stored in TransFix®-solution in this study could be explained by the use of leukocyte-spiked CSF samples. In these CSF samples, a cell content averaging 3.3302 × 10^6^/ml was set at day 0. Thus, the starting cell count on day 0 in the leukocyte-spiked CSF samples was many times higher than physiologically detected in CSF samples. In the study by Jongste et al. the average leukocyte content was 5 cells/μl with a range of < 1–552 cells/μl [8]. The high leukocyte count in the CSF samples used in this study made the differences between storage in the TransFix®-solution and untreated storage more apparent.

Previous authors reported that the neutrophilic granulocytes degenerate first [20, 24]. This was also seen in this flow cytometric study of leukocyte-spiked CSF samples. However, the CD21-positive lymphocyte population showed a significant decrease in the absolute number from day 0 to day 1, whereas the values on day 2 and 3 remained relatively constant. Therefore, this subpopulation of B-lymphocytes also appears to degenerate early during storage in CSF and is therefore in contrast to the remaining CD3- and CD4-positive T-lymphocytes. Among other things, flow cytometry in veterinary medicine is used to differentiate the phenotypes of leukocytes in the CSF more closely and thus to distinguish B- and T-cell lymphomas of the CNS [11]. In particular, the correct classification of a lymphoma is important, since the different phenotypes have different prognoses [13, 22] and the response to therapy is also different [13]. However, when using the TransFix®-stabilization solution, there was a significant decrease in the absolute number of CD21-positive B lymphocytes between day 0 and day 1 in the CSF samples in this study, which can result in a wrong phenotyping of a lymphoma.

Furthermore, de Jongste et al. found the proportion of lymphocytes in particular was significantly higher in the samples stored in TransFix®-solution [8]. While the number of monocytes was also higher in the samples stored in TransFix®-solution, no significant difference was found for the granulocytes between the untreated samples and the samples stored in TransFix®-solution [8]. Only a partial agreement with the results of de Jongste et al. could be demonstrated in this study. In contrast to de Jongste et al., a significantly higher number for the granulocytes was also measurable in the samples with TransFix®-solution compared to the untreated samples at all points in time. Nevertheless, a significantly higher number of lymphocytes were also detected in the samples stored in TransFix®-solution on all 3 days. Only for the antibody against CD21 on day 3 no significant difference could be determined as already described. No statement could be made about the monocytes due to the lack of measurability. According to de Jongste et al., the TransFix®-solution was insufficiently effective for the stabilization of monocytes and granulocytes [8]. In this study a successful stabilization of the granulocytes was also observed. However, the help of the histogram had to be used here for the evaluation, since a left shift of the entire population made the evaluation by means of histoplot difficult.

## Conclusion

In conclusion to our study results, the “TransFix®/EDTA CSF Sample Storage Tubes” can be used for extended storage prior to flow cytometric analysis especially of T-lymphocytes and granulocytes in canine and feline CSF samples. The detection of monocytes with the antibody against CD14 (clone TÜK14) was no longer possible after storage in the “TransFix®/EDTA CSF Sample Storage Tubes”. However, standard examinations, such as microscopic counting of leukocytes and erythrocytes as well as morphological cell assessment on cytospin preparations should be performed on fresh CSF samples within 30 min after sampling.

## Methods

The current study consists of two parts. In the first one microscopic count of leukocytes and erythrocytes and the morphologic assessment of cells on cytospin preparations was evaluated. In the second part of the study blood cell-spiked cerebrospinal fluid samples were analyzed by flow cytometry. In both parts the untreated storage was compared to the storage in TransFix® tubes.

### Samples

**Fig. 3:**
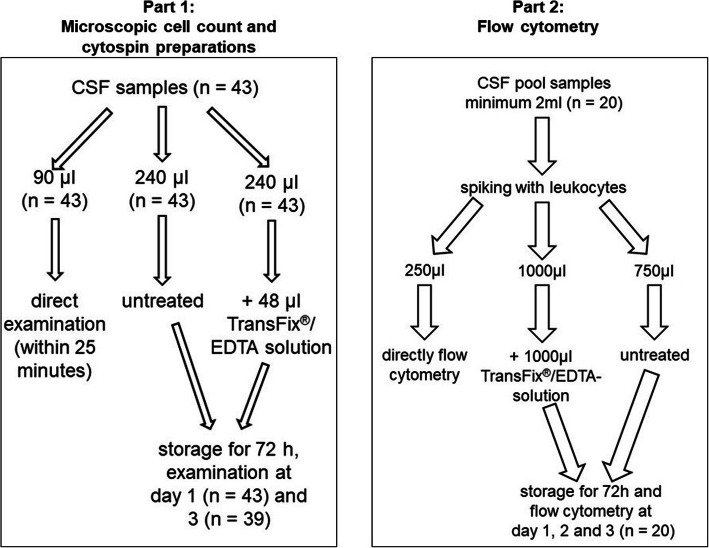
Distribution of the samples for the storage of part 1 and 2 of this study

All samples were taken at the Department of Small Animal Medicine and Surgery, University of Veterinary Medicine Hannover (Germany) between the 20 August 2018 and 4 April 2019. During the study period 43 fresh CSF samples, five feline and thirty-eight canine CSF samples, were analyzed for microscopic cell counting and examination of cell morphology. 22 samples were obtained as part of diagnostic tests in dogs and cats with neurological diseases with written owners’ consent and 4 samples were leftover from a granted animal experiment (animal experiment reference number: 33.8–42,502-05-18A290) from healthy dogs. 17 additional samples were obtained post mortem from patients immediately after euthanasia, due to CNS diseases in 7 cases and due to other diseases in the other 10 cases. The study was performed according to the ethical guidelines of the University and was approved by the Universities thesis Committee. All samples were obtained suboccipitally or lumbar in general anaesthesia or post mortem into plain tubes. Within a maximum delay of 25 min after sampling the examination of the untreated sample on day 0 started and the TransFix®-solution was added to the corresponding amount of the CSF sample as shown in Fig. 3. Furthermore 20 blood leukocyte-spiked canine CSF pool samples were analyzed by using flow cytometry and comparing untreated storage and storage in TransFix®-solution. The CSF samples, which were pooled and during the experiments spiked with leukocytes separated from canine EDTA blood samples, have been stored at a temperature of − 80 °C and their cell count, protein content (albumin and globulin) and glucose levels had been within the physiological range before freezing.

For the storage the fresh CSF samples respectively the blood-leucocyte spiked CSF pool samples were divided into three parts, as shown in Fig. 3. The untreated samples and the samples mixed with TransFix®-solution were stored in microcentrifugation tubes or in 5 ml plain tubes in the fridge for the whole study period. Fresh samples, the untreated stored samples and the samples stored in TransFix®-solution were examined with the same protocol at all time points of analysis. But in samples stored in TransFix®-solution a washing step with phosphate-buffered saline (PBS) had to be included. Therefore, PBS was added to the samples in TransFix®-solution and centrifuged for 5 min at 540×g according to the manufacturer’s instruction. Afterwards the supernatant was removed, and the cell pellet was resuspended in the same amount of PBS as in the original sample resulting in the original cell concentration.

#### Part 1: microscopic cell count and evaluation of cytospin preparations

For the examination 10 μl of Türk’s solution (PanReac AppliChem, Darmstadt, Germany) were mixed with 90 μl of the relevant CSF sample for staining the leucocytes. After 3 min of staining 20 μl of this mixture were transferred to a Fuchs-Rosenthal counting chamber (C-Chip™ Disposable Counting Chamber; NanoEnTek Inc., Seoul, Korea). Leucocytes and erythrocytes were counted via microscopic observation using the 400fold magnification and considering all visible leucocytes and erythrocytes in the squares of the counting chamber.

The remaining 80 μl of the mixture of CSF and Türk’s solution were mixed with 40 μl albumin (10% bovine serum albumin (BSA) [Albumin Fraktion V, Carl Roth GmbH + Co KG, Karlsruhe, Germany] in PBS) to prepare the cytospin using HETTICH-ZYTO technique (Hettich AG, Bäch, Switzerland). Cells were mounted on specimen slides (Superfrost® Plus, Thermo Scientific™, Thermo Fisher Scientific, Waltham, Massachusetts, USA) by centrifuging (HETTICH Rotina 35R; Hettich AG, Bäch, Switzerland) for 3 min at a speed of 1700 rounds per minute (rpm). For drying specimen slides were centrifuged for 1 min at a speed of 3400 rpm with a filter card (Hettich AG, Bäch Switzerland). Afterwards the preparation was stained using Papenheim coloring (Merck KGaA, Darmstadt, Germany). After drying preparations were covered permanently with Eukitt® Quick-hardening mounting medium (Sigma-Aldrich, Merck KGaA, Darmstadt, Germany) and a cover slip, so that the microscopic evaluation with a thousandfold magnification with the use of oil immersion was possible.

During the microscopic analysis of the cytospin preparations lymphocytes, segmented and rod-nucleated as well as eosinophilic granulocytes, monocytes, macrophages and blast cells were differentiated and the proportion of the cells was determined.

### Part 2: flow cytometry

#### Isolation peripheral blood leucocytes

Two and a half milliliter of a canine blood sample were mixed with 5 ml PBS and afterwards layered carefully on 2 ml Human Pancoll (PAN Biotech, Aidenbach, Germany) in another 15 ml tube (Sarstedt AG + Co KG, Nümbrecht, Germany). The falcon tube was centrifuged for 30 min at a speed of 1100×g and a temperature of 10 °C in the Hettich Rotina 35R centrifuge (Hettich, Bäch, Switzerland). This centrifuge was used in all following centrifugation steps. After centrifugation, the leukocytes were separated from the erythrocytes and were pipetted in another 15 ml tube with FACS staining buffer. The FACS staining buffer consists of autoMACS™ Rinsing Solution (MACS Miltenyi Biotec, Bergisch Gladbach, Germany) with 0.5% fetal calf-serum (Gibco®, Thermo Fisher Scientific, Carlsbad, USA). The new 15 ml tube was again centrifuged for 8 min at a speed of 500×g and at room temperature. Then the supernatant was removed and the pellet was resuspended in FACS staining buffer. In case of erythrocyte contamination of the pellet a lysis of the erythrocytes with distilled water was performed. The cells were washed with PBS and centrifuged two further times for 8 min at a speed of 200×g and a temperature of 20 °C. Afterwards the supernatant was removed completely and the pellet was resuspended in one milliliter of the canine CSF sample pool.

For the determination of the leukocyte content, a cell count was performed with 90 μl trypan blue (Sigma Aldrich, Steinheim, Germany) and ten microliters of the prepared cell-CSF-mixture. Both components were mixed transferred to a Neubauer improved counting chamber (C-Chip™ Disposable Counting Chamber; NanoEnTek Inc., Seoul, Korea) for microscopic leucocyte count. The cell content of the cell-CSF-mixture was adjusted to 4x10^6^cells/ml.

### Staining + flow Cytometry

The examination via flow cytometry was done on day 0 and on the three following days (day 1, 2 and 3) to compare the untreated storage and the storage in TransFix® tubes. 250 μl of the relevant sample were pipetted into a 3.5 ml tube. After blocking with 13.2 μl Trustain (Human TruStain FcX™, BioLegend, San Diego, USA) and thorough mixing, 50 μl of the prepared CSF cell mixture each were used for staining with antibodies and isotypes (Table 2). One part remained untreated and no antibody was added.

**Table 2:** Used antibodies for the flow cytometry

Tube	AB against	Name + Producer	Order-Number	Concentration/ Used Amount/ Dilution	Detected cells	Channel/ Laser
1	Untreated	ToProTM −3 Iodide, Life Technologies, Thermo Fisher Scientific, Carlsbad, USA	T3605		dead cells	R1/Red 635 nm
2	CD3 conjugated with FITC Clone CA17.2A12	Mouse anti dog CD3 FITC; Bio-Rad, Hercules, USA	MCA1774F	0,1 mg/ml 5 μl 11:1	T-lymphocytes [25]	B1/Blue 488 nm
2	CD4 conjugated with PE Cy7 Clone YKIX302.9	Anti-canine CD4 PE Cyanine 7; eBioscience, San Diego, USA	25–5040-42	12 μg/ml 5 μl 11:1	helper-T-cells [3, 25]	B2/Blue 488 nm
2	CD11b conjugated with PerCP Vio® 700 Clone REA592	CD11b PerCP-Vio® human and mouse; MACS Miltenyi Biotec, Bergisch Gladbach, Germany	130–109-289	30 μg/ml 5 μl 11:1	granulocytes, monocytes, NK-cells, subpopulation of T−/B-lymphocytes [19]	B2/Blue 488 nm
2	CD14 conjugated with PE Clone TÜK4	Mouse anti Human CD14:RPE; Bio-Rad, Hercules, USA	MCA1568 PE	ca. 0,1 mg/ml 7,1 μl 8,04:1	monocytes and macrophages, sporadic granulocytes [12]	B3/Blue 488 nm
2	CD45 conjugated with AF 647 Clone YKIX 716.13	Rat anti Dog CD45 Alexa Fluor 647; SeroTec, Hercules, USA	MCA1042A647	0,05 mg/ml 5 μl 11:1	all leucocytes in peripheral blood [6]	R1/Red 635 nm
3	Isotype CD 3 FITC Clone W3/25	Mouse IgG1 negative control FITC; SeroTec, Hercules, USA	MCA928F	0,1 mg/ml 5 μl 11:1		B1/Blue 488 nm
3	Isotype CD4 PE Cy7 Clone eBR2a	Rat IgG2a kappa Isotype Control PE Cyanine 7; eBioscience, San Diego, USA	25–4321-82	0,2 mg/ml 5 μl 11:1		B2/Blue 488 nm
3	Isotype CD11b PerCP Vio® 700 Clone REA293	REA control PerCP Vio 700 human; MACS Miltenyi Biotec, Bergisch Gladbach, Germany	130–113-441	5 μl / 11:1 (new load: 1 μl / 51:1)		B2/Blue 488 nm
3	Isotype CD14 PE	PE Mouse IgG2a, κ isotype Ctrl; BioLegend, San Diego, USA	400,214	7,1 μl 8,04:1		B3/Blue 488 nm
3	Isotype CD45 AF 647	RAT IgG2b negative Contral AlexaFluor 647; SeroTec, Hercules, USA	MCA1125A647	5 μl 11:1		R1/Red 635 nm
4	CD21 conjugated with PE Clone CA2.1D6	Mouse anti canine CD21:RPE; Bio-Rad, Hercules, USA	MCA1781PE	0,05 mg/ml 5 μl 11:1	B-lymphocytes	B4/Blue 488 nm
5	Isotype CD21 PE Clone W3/25	Mouse IgG1 negative Control:RPE; Bio-Rad Hercules, USA	MCA928 PE	5 μl 11:1		B4/Blue 488 nm

Used Antibodies (AB) and Expression of the biomarker. (*FITC* Fluoresceinisothiocyanat, *PE Cy7* Phycoerythrin Cyanine7, *AF* Alexa Fluor, *PE* Phycoerythrin, *NK-cell* Natural killer cell)

After a time of 20 min for incubation in the fridge 500 μl FACS staining buffer were added to each tube and all tubes were centrifuged again for 8 min at a centrifugal force of 300×g. After the decantation of the supernatant the cell pellets were resuspended in 200 μl FACS staining buffer with the exception of the tubes with the number 1 of the untreated CSF-samples. The cell pellets of the tubes with the number 1 of the untreated samples were resuspended in 200 μl 1 μM ToPro-solution (ToProTM − 3 Iodide, Life Technologies, Thermo Fisher Scientific, Carlsbad, USA) in FACS staining buffer for differentiating dead and living cells. Immediately after pipetting the flow cytometric examination was started using the MACSQuant Analyzer 10 (Miltenyi Biotec, Bergisch Gladbach, Germany).

### Evaluation of the results

The results were analyzed using the MACSQuantify™ Software (MACS Miltenyi Biotec; Bergisch Gladbach, Germany). For the examination of the lymphocytic population the antibodies against CD3, CD4, CD21 and CD45 were used. The antibodies against CD11b and CD45 served for analysis of the granulocytes and the population of monocytes was separately examined using the antibody against CD14. The cut-off in the histoplot of each isotype was set at 2% (+/− 0.2%). For the evaluation of the CD11b positive populations and some of the CD45 positive populations a histogram was used in addition and the cut-off for positive cells was set at the point of intersection between the histograms of the isotype and the sample.

### Statistical analysis

Statistical evaluation was carried out with the SAS Enterprise Guide 7.1. (SAS Institute Inc., Cary, North Carolina, USA). Descriptive statistics were used to examine the results of the microscopic cell count and the evaluation of the cytospin preparations. The results of the flow cytometric assay were analyzed by descriptive statistics and the mixed model Analysis of Variance (ANOVA). For each antibody, an evaluation was made separately for the percentages as well as for the absolute numbers of positive cells for the respective antibody. If the values ​​of the studentized residuals for the parameter to be examined deviated significantly from the normal distribution, the values ​​were transformed with the decadic logarithm and then the evaluation was repeated. For the post-hoc tests the correction according to Turkey-Kramer was used. The previously logarithmized values ​​were subsequently transformed back, so that in this publication only the inverse transformed values ​​are given. The limit for an existing significance was an adjusted *p*-value (Adj P) of <.05.

Supplementary Material: Results Part 1.

Results of the evaluation of the cytospin preparations compared between day 0, day 1 and day 3 of untreated samples and samples stored in TransFix®-solution.

## Supplementary Information

**Additional file 1.**

## Data Availability

The datasets generated, used and analysed during the current study are available from the corresponding author on reasonable request.

## Abbreviations

AB: Antibody
ANOVA: Analysis of Variance
CNS: Central Nervous System
CSF: Cerebrospinal Fluid
EDTA: Ethylenediaminetetraacetic Acid
RBC: Red Blood Cells
TNC: Total Nucleated Cells
WBC: White Blood Cell
